# Differential Diagnosis, Prevention Measures, and Therapeutic Interventions for Enhanced Monkeypox (Mpox) Care

**DOI:** 10.7759/cureus.60724

**Published:** 2024-05-20

**Authors:** Imran Khan, Mahalakshmi S, Tanu Dixit, Rohan Shinkre, Selvan Ravindran, Sukanta Bandyopadhyay

**Affiliations:** 1 Medical Surgical Nursing, Sharda School of Nursing Science and Research, Sharda University, Greater Noida, IND; 2 Community Health Nursing, Cheran College of Nursing, Tamil Nadu Dr. M.G.R. Medical University, Coimbatore, IND; 3 Symbiosis School of Biological Sciences, Symbiosis International (Deemed University), Pune, IND; 4 Central Research Wing, KLE Society’s Institute of Dental Sciences, Bengaluru, IND; 5 Symbiosis School of Biological Sciences, Faculty of Medical and Health Sciences, Symbiosis International (Deemed University), Pune, IND; 6 Biochemistry, Rama Medical College Hospital & Research Centre, Kolkata, IND

**Keywords:** poxvirus infections, zoonotic diseases, nursing care, monkeypox, etiology, differential diagnosis

## Abstract

Monkeypox (Mpox) is a rare viral disease that presents considerable challenges in healthcare settings, necessitating enhanced nursing care for effective management. This review thoroughly explores key aspects related to improving nursing care for Mpox. It commences by examining the background information on Mpox, encompassing its etiology, epidemiology, and modes of transmission. The differential diagnosis of Mpox is investigated, elucidating its clinical presentation, symptoms, and diagnostic methods to differentiate it from similar conditions. Prevention and control measures at both the public health and healthcare levels are scrutinized, including surveillance and reporting, contact tracing, isolation, and vaccination programs. In healthcare settings, infection prevention and control strategies, such as proper utilization of personal protective equipment, hand hygiene, and environmental management, are discussed. Furthermore, therapeutic interventions for Mpox, including symptomatic management, antiviral therapy, and supportive care, are outlined, with a specific emphasis on pain management, fever control, and psychosocial support. Nursing care strategies encompass patient assessment and monitoring, infection prevention strategies, psychosocial support, and patient education. The challenges encountered in enhancing nursing care for Mpox are acknowledged, along with research gaps and areas for further investigation. Finally, innovations in nursing practice for improved care, such as technology integration and simulation-based training, are explored. Enhancing nursing care in Mpox is crucial for positive patient outcomes, reducing transmission risks, and promoting overall well-being. By addressing the unique challenges, conducting further research, and embracing innovative practices, healthcare professionals, particularly nurses, can provide optimal care and contribute to better management of Mpox cases.

## Introduction and background

Monkeypox (Mpox), a rare zoonotic disease caused by the monkeypox virus (MPXV), has gained attention due to its potential public health impact and its similarities to human smallpox. While primarily endemic in Central and West African regions, recent cases outside these areas have heightened global concerns. This review explores the crucial aspects of Mpox, focusing on the differential diagnosis, prevention measures, and therapeutic interventions that collectively contribute to enhanced care for affected individuals.

Mpox poses a significant diagnostic challenge due to its clinical resemblance to other orthopoxviruses, such as smallpox and variola virus. The need for accurate and rapid differential diagnosis is paramount to guide appropriate treatment and public health responses. Molecular techniques, including polymerase chain reaction (PCR) assays, play a pivotal role in distinguishing Mpox from similar diseases. Serological assays, such as enzyme-linked immunosorbent assays (ELISAs), aid in detecting specific antibodies, further refining the diagnosis. Additionally, advances in diagnostic imaging techniques, such as magnetic resonance imaging (MRI), contribute valuable insights into the disease pathogenesis and aid in distinguishing Mpox from other viral infections. Efficient prevention strategies are essential for mitigating the spread of Mpox. Vaccination remains a cornerstone in preventing orthopoxvirus infections. Although the smallpox vaccine provides partial cross-protection, the development of a specific Mpox vaccine has been pursued to enhance efficacy and reduce adverse effects associated with the existing vaccine. Additionally, implementing strict control measures in animal markets and promoting public awareness about the disease’s zoonotic nature are crucial for preventing spillover events. Studies have emphasized the importance of a comprehensive One Health approach, involving collaboration between human and animal health sectors, to effectively control Mpox transmission. Therapeutic interventions for Mpox focus on alleviating symptoms, reducing complications, and limiting virus transmission. Antiviral medications, such as cidofovir and brincidofovir, have shown promise in reducing mortality and morbidity associated with Mpox. However, the optimal timing and duration of antiviral therapy require further investigation. Supportive care, including fluid management and pain control, remains essential for improving patient outcomes. In severe cases, where respiratory distress or other complications arise, advanced medical interventions such as mechanical ventilation may be necessary. The development of targeted therapeutic agents against specific viral proteins is an active area of research, holding promise for more effective treatment options in the future [1-7].

## Review

Differential diagnosis

Clinical Manifestations of Monkeypox

The clinical manifestations of Mpox encompass a spectrum of symptoms that overlap with various poxvirus infections, necessitating a thorough understanding for accurate diagnosis and management. The prodromal phase typically manifests with non-specific symptoms such as fever, headache, and myalgia, which can easily be mistaken for common viral infections [1]. However, the subsequent development of characteristic vesicular skin lesions is a hallmark feature of Mpox, aiding in its differentiation from other illnesses [2].

Diagnostic strategies play a pivotal role in confirming Mpox infection and guiding appropriate clinical management. Molecular techniques, particularly PCR assays, have revolutionized the detection of MPXV DNA, offering high sensitivity and specificity [3]. These assays enable rapid identification of the virus, facilitating prompt initiation of treatment and implementation of infection control measures. Furthermore, serological assays, such as ELISAs, serve as valuable tools for confirming Mpox infection by detecting specific antibodies in patient samples [4].

In addition to molecular and serological assays, diagnostic imaging modalities contribute significantly to the accurate diagnosis of Mpox. MRI, in particular, provides detailed insights into the pathogenesis of the disease, allowing clinicians to distinguish Mpox from other viral infections based on characteristic imaging findings [5]. The use of MRI in conjunction with clinical and laboratory findings enhances diagnostic accuracy and enables tailored therapeutic interventions.

While diagnostic approaches have advanced significantly in recent years, challenges remain in differentiating Mpox from other poxvirus infections and similar clinical syndromes. Further research is warranted to explore novel diagnostic markers and imaging techniques that can enhance the specificity and sensitivity of diagnostic assays, ultimately improving patient outcomes and guiding public health interventions.

Distinguishing Features From Other Poxvirus Infections

Distinguishing Mpox from other poxvirus infections presents a significant challenge due to overlapping clinical manifestations. However, advancements in diagnostic techniques have enabled the identification of unique features specific to Mpox, enhancing diagnostic precision.

Molecular diagnostic techniques, notably PCR assays, have revolutionized the identification of MPXV by targeting specific genetic markers [1]. These assays offer high sensitivity and specificity, allowing for rapid and accurate detection of Mpox infection. By amplifying and analyzing viral DNA, PCR assays enable clinicians to differentiate Mpox from other poxviruses, facilitating targeted therapeutic interventions and infection control measures.

Serological assays, such as ELISAs, complement molecular techniques by detecting specific antibodies produced in response to Mpox infection [5]. The presence of these antibodies serves as a reliable indicator of Mpox infection, further confirming the diagnosis and guiding appropriate clinical management. ELISAs offer a non-invasive and cost-effective means of serodiagnosis, particularly in resource-limited settings where PCR may not be readily available.

Diagnostic imaging, particularly MRI, provides additional insights into the characteristic lesions associated with Mpox, aiding in differentiation from other poxviruses [4]. MRI enables clinicians to visualize the extent and distribution of lesions, facilitating accurate diagnosis and assessment of disease severity. By identifying specific imaging features unique to Mpox, MRI contributes valuable information to the diagnostic process, guiding therapeutic interventions and prognostication.

In conclusion, a multifaceted diagnostic approach incorporating molecular techniques, serological assays, and diagnostic imaging is essential for accurately distinguishing Mpox from other poxvirus infections. By leveraging the unique features of Mpox, clinicians can implement targeted therapeutic interventions and public health measures, ultimately improving patient outcomes and preventing further transmission of the virus.

Role of Molecular and Serological Diagnostic Tools

The integration of molecular and serological diagnostic tools plays a pivotal role in advancing the differential diagnosis of Mpox and optimizing patient care. Molecular techniques, such as PCR assays, demonstrate high specificity in detecting MPXV DNA, facilitating early and accurate identification of the infection [1]. Additionally, serological assays, including ELISAs, contribute to the diagnostic arsenal by detecting specific antibodies produced during infection [5]. The synergistic application of these tools enables a comprehensive understanding of the viral dynamics, aiding in distinguishing Mpox from other orthopoxviruses. Molecular diagnostics offer rapid detection, crucial for timely clinical decision-making, while serological assays enhance sensitivity and contribute to retrospective case identification. The combination of these diagnostic modalities is essential for a nuanced and effective approach to patient management, allowing for prompt initiation of appropriate therapeutic interventions and implementation of preventive measures. This review explores the working mechanisms and effectiveness of molecular and serological tools in the context of Mpox, emphasizing their significance in enhancing diagnostic precision and subsequently improving overall patient care.

Epidemiology and global distribution

Geographical Prevalence and Hotspots

Mpox, a zoonotic orthopoxvirus infection, exhibits distinct geographical prevalence patterns and hotspots that play a pivotal role in understanding and managing its transmission dynamics. This review synthesizes current literature to elucidate the geographic distribution of Mpox, identifying regions with higher incidence rates and potential hotspots for zoonotic spillover events. Employing spatial analysis methods, such as geographic information systems and remote sensing, facilitates the identification of environmental factors contributing to the persistence and transmission of Mpox. The review also highlights the relevance of epidemiological surveillance systems in monitoring the spatial dynamics of Mpox outbreaks. A comprehensive examination of geographical prevalence and hotspots is critical for tailoring targeted prevention measures and intervention strategies, ultimately contributing to enhanced Mpox care. Several studies [1,2,6] provide insights into the spatial distribution, zoonotic aspects, and preventive measures associated with Mpox.

Historical Trends and Notable Outbreaks

Through their observation over time and documentation of previous Mpox outbreaks, we can get a vital insight into the changing dynamics and evolution of this disease that is caused by animals. The analysis of Mpox patterns witnessed from documented cases through time proves the need for regionally tailored prevention strategies. Major outbreaks, the latter including the U.S. 2003 case, Centers for Disease Control and Prevention (2003), serve as examples of how easily a disease can spread internationally, thus making the presence of a robust system of surveillance all the more necessary. Hence, digging through history explains how Mpox is adaptable and highlights the essence of some complex factors related to the outbreak and transmittance. This review integrates highlights from studies [1,2,4] to discuss the importance of having an insight into the historical perspectives whereby it helps to come up with informed measures that apply to prevention and control currently as well as in the future.

Factors Influencing the Spread of Monkeypox

The global spread of Mpox is influenced by a multitude of factors, necessitating a comprehensive examination to enhance preventive strategies. This review investigates the ecological, epidemiological, and host-related determinants that contribute to the dissemination of Mpox. Ecologically, factors such as land use changes, deforestation, and urbanization impact the habitat of potential animal reservoirs, influencing the risk of zoonotic transmission to humans [2]. Additionally, the epidemiological landscape, including population density and international travel, plays a pivotal role in shaping the dynamics of Mpox outbreaks [4]. Furthermore, host-related factors, including immunity levels and genetic susceptibility, contribute to the variation in individual responses to the virus, influencing transmission dynamics within human populations [1]. Understanding these factors is critical for designing targeted prevention measures to curtail the global spread of Mpox and mitigate its public health impact.

Viral pathogenesis

Molecular Characteristics of Monkeypox Virus

Investigating the viral genome, comprising linear double-stranded DNA, reveals key genes associated with virulence and host interaction, such as the M- and F-box proteins, critical for immune evasion and replication [8]. Understanding the virus’s molecular structure is essential for developing targeted diagnostic tools and therapeutic interventions. Furthermore, the glycoproteins on the viral envelope, particularly the monkeypox hemagglutinin, play a crucial role in viral entry, making them potential targets for antiviral drug development [9]. This review emphasizes the significance of unraveling the molecular landscape of MPXV for advancing diagnostic accuracy.

Mechanisms of Viral Entry, Replication, and Spread Within the Host

This review investigates the intricate mechanisms underlying the viral entry, replication, and dissemination of MPXV within the host organism. Upon initial host cell attachment, the virus employs specific molecular interactions, facilitated by viral surface proteins, to gain entry into the host cell. Following entry, the virus undergoes uncoating, liberating its genetic material into the cellular milieu. The subsequent stages involve viral transcription and translation processes, leading to the synthesis of viral structural and non-structural proteins. Viral replication occurs within discrete cellular compartments, contributing to the assembly of mature virions. The dissemination of progeny virions involves intricate interactions with host cell machinery, facilitating their release and subsequent infection of neighboring cells. Understanding these molecular intricacies is paramount for the development of targeted therapeutic interventions to impede viral replication and enhance host immune responses. This review synthesizes current knowledge on these mechanisms, shedding light on potential therapeutic targets and advancing strategies for enhanced Mpox care.

Prevention measures

Overview of Existing Preventive Strategies

This review provides a comprehensive overview of existing preventive strategies for Mpox, emphasizing their mechanisms, efficacy, and scientific foundations. The primary preventive measure against Mpox involves vaccination, with the smallpox vaccine demonstrating partial cross-protection. It is suggested that this vaccination may offer a degree of immunity against Mpox [6]. However, ongoing research focuses on developing a specific Mpox vaccine to enhance efficacy and minimize potential adverse effects associated with the smallpox vaccine. Furthermore, a One Health approach, integrating human and animal health efforts, is crucial in preventing zoonotic transmission. Control measures in animal markets and public awareness campaigns are integral components of this strategy [2]. This review provides a concise exploration of the current state of preventive strategies, addressing vaccination, One Health initiatives, and associated research directions for improved Mpox.

Efficacy of Smallpox Vaccination

The efficacy of smallpox vaccination in the prevention and control of Mpox is a critical aspect of zoonotic disease management. Smallpox vaccination, which induces immunity through the introduction of the vaccinia virus, has shown cross-protection against Mpox due to the antigenic similarities between the two viruses [6]. The vaccine primarily functions by eliciting a robust cellular and humoral immune response, targeting specific viral proteins, and preventing viral replication within the host. This review explores the molecular and immunological mechanisms underlying the smallpox vaccine’s effectiveness against Mpox, emphasizing its role in reducing the severity of clinical manifestations and limiting viral transmission. Additionally, the discussion encompasses the challenges and considerations in implementing smallpox vaccination as a preventive measure in regions endemic to Mpox, contributing to a comprehensive understanding of its efficacy in enhancing Mpox care.

Development and Progress of Specific Monkeypox Vaccines

The development of specific Mpox vaccines represents a critical stride in mitigating the impact of this zoonotic orthopoxvirus. Current research focuses on refining and augmenting existing smallpox vaccination strategies, given their partial cross-protection. Novel vaccine candidates, such as the live attenuated modified vaccinia ankara vector expressing Mpox antigens, have shown promising results in preclinical studies, eliciting robust immune responses and conferring protection against virulent Mpox challenge [10]. Additionally, advancements in viral vector-based vaccines, including recombinant vesicular stomatitis virus expressing Mpox glycoproteins, are being explored for their ability to induce protective immunity [11]. These vaccines leverage genetic engineering techniques to elicit a targeted immune response, enhancing specificity and reducing adverse effects associated with traditional vaccination methods. As research progresses, the development of a specific Mpox vaccine holds the potential to revolutionize preventive measures, offering a tailored and efficacious solution in the global fight against this emerging infectious disease.

Public health strategies

Surveillance Systems for Early Detection

Effective surveillance systems are critical for the early detection of Mpox outbreaks, providing a foundation for timely public health responses. The surveillance mechanism involves a multidisciplinary approach, integrating clinical, laboratory, and epidemiological data. Molecular diagnostic tools, such as PCR assays, play a pivotal role in swiftly confirming Mpox cases [1]. Sentinel surveillance sites strategically positioned in endemic regions facilitate the monitoring of febrile illnesses, enabling rapid identification of potential Mpox cases. Additionally, syndromic surveillance, focusing on specific clinical symptoms associated with Mpox, aids in the early recognition of outbreaks [5]. The integration of data from animal health surveillance systems, emphasizing potential zoonotic spillover events, further enhances the sensitivity of the surveillance network. This systematic approach ensures the timely implementation of control measures, such as isolation and contact tracing, minimizing the impact of Mpox on both individual health and public safety.

Public Awareness Campaigns and Education

Public awareness campaigns and education are essential components in the efforts to mitigate the impact of Mpox by disseminating vital information to at-risk populations. These campaigns utilize various communication channels, encompassing both traditional and digital media platforms, to effectively reach diverse audiences. By drawing on scientific insights and evidence-based strategies, these initiatives aim to enhance the understanding of Mpox transmission dynamics, symptoms, and preventive measures among the public. Tailored educational materials, such as brochures and online resources, play a crucial role in increasing knowledge and awareness within communities.

The effectiveness of these campaigns is evaluated by assessing changes in knowledge, attitudes, and behaviors related to Mpox prevention and care. Additionally, community engagement is emphasized to foster a sense of shared responsibility among individuals, empowering them to adopt protective measures and actively participate in surveillance efforts. A well-designed awareness campaign not only serves to educate the public but also cultivates a proactive and informed community. In doing so, these campaigns contribute significantly to the overall success of Mpox prevention and control strategies [12,13].

## Conclusions

The amalgamation of precise differential diagnosis, targeted prevention measures, and innovative therapeutic interventions constitutes a paramount framework for advancing the care of Mpox patients. The utilization of molecular techniques, such as PCR assays, has proven instrumental in discerning the distinct characteristics of Mpox, enabling prompt and accurate identification amidst the clinical landscape of orthopoxviruses. These diagnostic modalities, coupled with serological assays such as ELISAs, play a pivotal role in enhancing diagnostic specificity. Prevention strategies, underscored by vaccination initiatives, exhibit notable efficacy in conferring partial cross-protection against Mpox, accentuating the importance of continued research in developing dedicated Mpox vaccines for heightened prophylactic efficacy. Moreover, the implementation of a comprehensive One Health approach, integrating human and animal health sectors, emerges as a robust preventive measure, curbing zoonotic transmission. Therapeutically, the deployment of antiviral medications, including cidofovir and brincidofovir, showcases promising outcomes in mitigating the morbidity and mortality associated with Mpox infections. However, the optimal timing and duration of antiviral therapy necessitate further elucidation. Supportive care measures, encompassing fluid management and pain control, synergistically contribute to comprehensive therapeutic interventions. Noteworthy advancements in diagnostic technologies, ongoing research in vaccine development, and the exploration of novel antiviral agents underscore the dynamic landscape of Mpox research, indicating a promising trajectory for the enhancement of care strategies in the future. In essence, the symbiotic integration of cutting-edge diagnostics, robust prevention paradigms, and targeted therapeutics fosters a holistic and efficacious framework for the augmented management of Mpox cases.
