# Correction: Differential Diagnosis, Prevention Measures, and Therapeutic Interventions for Enhanced Monkeypox (Mpox) Care

**DOI:** 10.7759/cureus.c184

**Published:** 2024-07-09

**Authors:** Imran Khan, Mahalakshmi S, Tanu Dixit, Rohan Shinkre, Selvan Ravindran, Sukanta Bandyopadhyay

**Affiliations:** 1 Medical Surgical Nursing, Sharda School of Nursing Science and Research, Sharda University, Greater Noida, IND; 2 Community Health Nursing, Cheran College of Nursing, Tamil Nadu Dr. M.G.R. Medical University, Coimbatore, IND; 3 Symbiosis School of Biological Sciences, Symbiosis International (Deemed University), Pune, IND; 4 Central Research Wing, KLE Society’s Institute of Dental Sciences, Bengaluru, IND; 5 Symbiosis School of Biological Sciences, Faculty of Medical and Health Sciences, Symbiosis International (Deemed University), Pune, IND; 6 Biochemistry, Rama Medical College Hospital & Research Centre, Kolkata, IND

This article has been corrected at the request of the submitting author to change his affiliation as follows:

Imran Khan Original: Narayan Nursing College, Gopal Narayan Singh University Medical Surgical Nursing Sasaram, IND Corrected: Sharda School of Nursing Science and Research, Sharda University, Medical Surgical Nursing, Greater Noida, IND

The authors deeply regret that these errors were not identified and addressed prior to publication.

 

